# Finders Keepers: A Case of the Rogue Pacing Wire

**DOI:** 10.7759/cureus.9823

**Published:** 2020-08-17

**Authors:** Asmara A Malik, Jahanzeb Malik

**Affiliations:** 1 Public Health, National University of Medical Sciences, Rawalpindi, PAK; 2 Cardiology, Rawalpindi Institute of Cardiology, Rawalpindi, PAK

**Keywords:** cardiac pacing, rv rupture, pacing lead, temporary pacemaker, right ventricular perforation

## Abstract

We report a middle-aged woman with frequent episodes of pre-syncope due to complete heart block. She had no previous history of anticoagulation or steroid therapy, and underwent temporary pacemaker insertion. In spite of only mild symptoms, she was found to have a right ventricle perforation on CT. After placement of permanent lead, the patient was discharged home in a stable condition with no further complaints on follow-up.

## Introduction

Cardiac perforation by a pacing lead is a rare but life-threatening complication of pacemaker implantation usually presents within 24 hours of implantation and is uncommon after that [1]. This complication tends to be more frequent when the tip of the pacing lead is placed at the right ventricular (RV) apex rather than the ventricular septum. We present the case of a 65-year-old woman with RV perforation by the temporary pacing wire. It was removed surgically, and a permanent pacemaker was inserted thereafter. She remained stable at one-month follow-up.

## Case presentation

**Figure 1:**
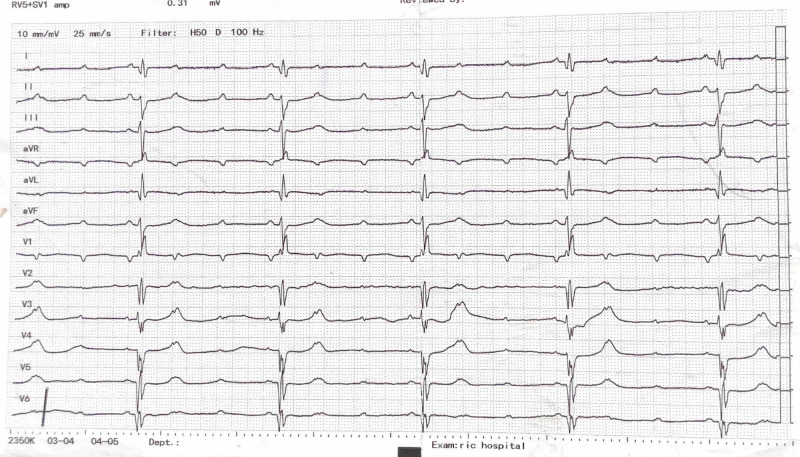
Electrocardiogram showing third-degree heart block

A 65-year-old female patient was admitted to our institute with frequent episodes of pre-syncope and a history of fall a day back. History was insignificant for chest pain, metabolic disease, or a rate-limiting drug. She had no comorbid conditions like diabetes, chronic kidney disease (CKD), or hypertension. She had a heart rate of 35 beats/min and a blood pressure of 100/60 mmHg. Her electrocardiogram (ECG) showed a third-degree atrioventricular (AV) block (Figure 1).

After taking consent from the attendant, a temporary pacemaker wire (SwanGanz™ Bipolar Pacing Catheter, Edwards Lifesciences Corporation, Irvine, CA, USA) was inserted via right femoral access and fixed at the RV apex after confirmation of pacing spike and a left bundle branch morphology on the cardiac monitor. It was fixed and the patient was comfortably moved back in electrophysiology bay. Her hematology, biochemistry, and metabolic profile were normal.

**Figure 2:**
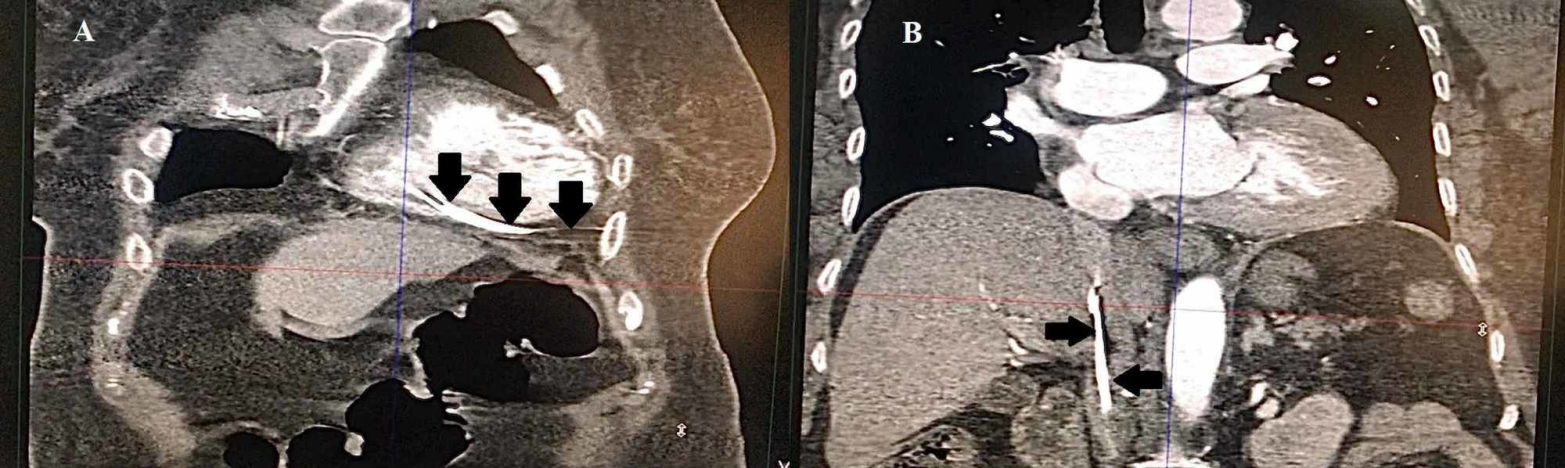
(A, B) Frontal plane of CT scan chest and abdomen showing the pacing wire (black arrows) coming via the inferior vena cava and going through the right ventricle into the thorax and abdominal cavity.

**Figure 3:**
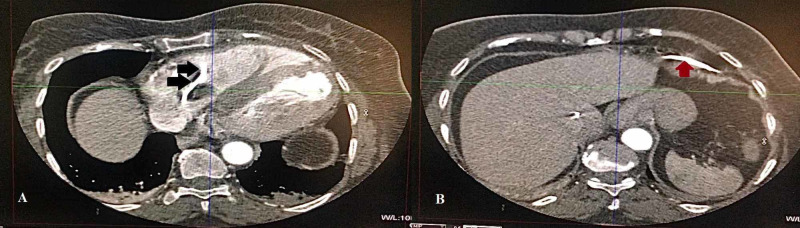
(A, B) An axial plane of CT showing the temporary pacing wire (black and red arrows) going through the right ventricle and into the abdominal cavity.

Echocardiography showed no structural heart disease or regional wall motion abnormality. She was being worked up for a permanent pacemaker. On the second day, she developed epigastric pain. She was treated for acid peptic disease after ruling out ischemia on ECG. Additionally, troponin I was done, which was normal. On the third day, her pain was persistent and she was intermittently pacing on the cardiac monitor. Thinking it as the irritation of the diaphragm by the pacing wire, she was moved under fluoroscopy to adjust her temporary wire. The operator was unable to move the lead and she was shifted back and her CT was ordered that showed perforation of the RV and the pacing lead in the abdominal cavity (Figures 2, 3).

A heart team was called, and it was decided to surgically remove the lead after discussing with the patient and taking her attendants consent. The pacing wire was safely removed, and postoperative echocardiography was repeated to look for pericardial effusion. It was normal. She was implanted a permanent dual-chamber pacemaker (CapSureFix Novus®, Medtronic, Minneapolis, MN, USA).

After a one-month follow-up, she was feeling well. There were no adverse or unanticipated events noted on follow-up. Echocardiography was normal, and pacing lead was visualized and normal.

## Discussion

Pacemaker insertion is indicated for bradycardia in patients not otherwise amenable to pharmacological intervention. With poor cardiac function, a myriad of symptoms such as fatigue, dizziness, and chest pain may occur, similar to our patient’s initial presentation. Pacemaker insertion is considered a relatively safe procedure with a perforation prevalence of only 0.1%-0.8%, found in the literature [2,3]. Lead perforation can be classified as acute (0-7 days), subacute (7-29 days), or delayed (≥30 days).

Their reported incidence in literature is highest for acute with 1%-7%, 1% for subacute, and 0.1% for delayed [4]. Our patient falls in the acute category. Most lead perforation tends to be atrial because of the thinner diameter of the atrial wall (2 mm) but our case was rare in being a ventricular type, even though the ventricular wall thickness is on average approximately 4 mm [3]. RV is reported to be the second most common site of perforation with a 6% incidence reported in the literature [5]. Some of the most frequently reported predictors of lead perforation are temporary leads, steroid use, active fixation leads, low body mass index (less than 20 kg/m^2^), older age, female gender, and concomitant anticoagulation therapy.

Operator experience has been found to be a possible cause of lead perforation but overtorquing or leaving too much loop cannot be ruled out in spite of the inserting physicians’ years of experience. Manufacturers’ recommendations for leads also vary across brands which could also lead to unintended non-compliance by the inserting physician [6]. 

Out of these, our case is unique in having a relatively younger (65 years) female patient than found in literature, with no previous history of anticoagulation or steroid therapy reported. Symptoms of perforation in patients vary from being asymptomatic to cardiac tamponade and death. A key learning point from our case and other reported mildly symptomatic cases is that normal pacemaker function does not exclude this complication.

Asymptomatic cases tend to be more in RV perforations as the right heart is a low-pressure system and can contain the sequelae of a perforation by muscle contraction and fibrosis over the lead [3]. Our patient also reported only persistent epigastric discomfort not amenable to pharmacologic treatment. By having high clinical vigilance and performing a CT scan, considered the gold standard in the diagnosis of cardiac perforation, an acute perforation can be corrected in time and prevent further complications like infections and cardiac tamponade from setting in [7].

## Conclusions

RV perforation is a rare but potentially fatal complication of pacemaker implantation. It usually manifests within 24 hours, but our patient developed it on the second day after the placement of the temporary pacing wire. This case emphasizes that clinical vigilance must be exercised in all patients who undergo lead placement to prevent further complications in patients.
